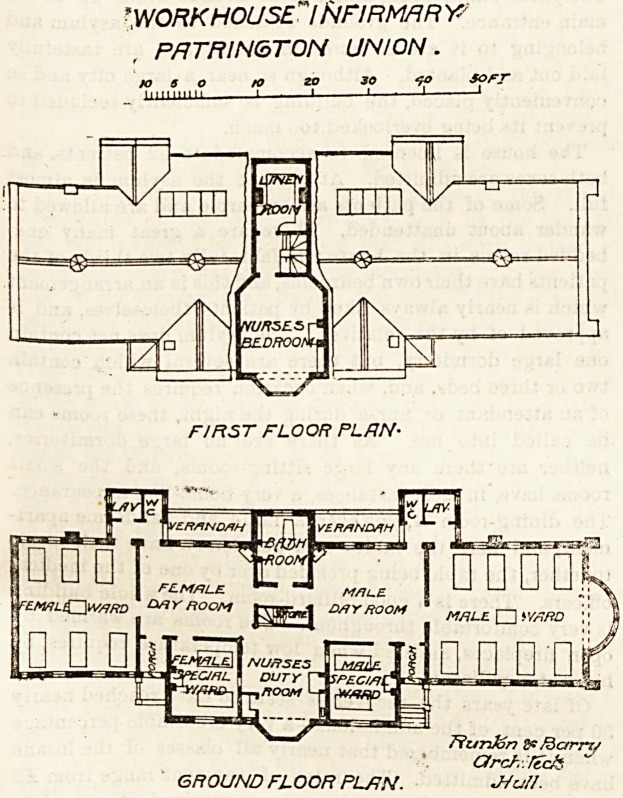# The Workhouse Infirmary, Patrington Union, East Yorkshire

**Published:** 1903-09-12

**Authors:** 


### THE WORKHOUSE INFIRMARY, PATRINGTON UNION, EAST YORKSHIRE.

THIS is a small infirmary containing only 18 beds, and might therefore be described as a cottage hospital. It follows the usual arrangement of a centre and two wings. The former contains the day-rooms, nurses'-room, special wards for two beds each, and a bath-room. The bath-room is so placed that it can be used by either men or women; and it projects southwards 5 or 6 feet, thus separating the men's verandah from the women's. Each day-room is lighted by two windows and a dormer. The nurses' duty-room occupies the centre of the north elevation, and has the male special ward on one side and the female on the other, both being commanded from the nurses'-room by observation windows. There are two entrances, one for each sex, on the north side. The whole of this section has been carefully thought out, and must be pronounced successful except that the special wards present the old difficulty of inadequate cross-ventilation. These rooms each contain 168 superficial feet of floor-space, that is 84 feet per bed, which is hardly enough for any special case, or indeed for many ordinary cases, as with 12-feet ceilings the space would work out at 1,008 cubic feet per bed. It would have been a very great improvement to draw these rooms 6 feet northwards, and so provide them with end bay-windows and with side windows too. The nurses'-room would be shorn of its bay, and the general elevation would not be so pleasing; but in planning a hospital efficiency of internal arrangements should come first. The male dormitory contains eight beds, and each bed is properly provided with a window on both sides. In addition there is a fine circular window at the west end. The female dormitory is for six beds, and the arrangements are similar, except that the circular bay is absent; an unaccountable omission it seems to us. The lavatory and closet blocks are well cut off from the main by cross-ventilating passages; and they are so arranged that they can be approached from either the day-room or the dormitory. It is evident that the architects have much ability in the planning of small hospitals, and this makes us more regret the faults in the small special wards, as otherwise the infirmary would have been for its size an extremely good one. Even with these drawbacks it is distinctly above the average.

The building is carried out in red stock bricks, and is covered with Wolliscroft tiles. The floors are of maple. Twyford's sanitary fittings are used. The architects were Messrs. Runton and Barry, of Hull, and Mr. Sergeant was contractor. The cost was something over £2,000, and this would be about £112 per bed, a sum by no means extravagant nowadays; but it must be remembered that the infirmary is merely an adjunct to an existing institution, hence requires no administrative sections.

## Figures and Tables

**Figure f1:**